# Implementation and evaluation of a palliative care training unit for EMS providers

**DOI:** 10.3389/fped.2023.1272706

**Published:** 2023-09-27

**Authors:** Holger Hauch, Naual El Mohaui, Michael Sander, Georg Rellensmann, Daniel Berthold, Peter Kriwy, Boris Zernikow, Julia Wager, Emmanuel Schneck

**Affiliations:** ^1^ Department of Children’s Pain Therapy and Pediatric Palliative Care, Faculty of Health, School of Medicine, Witten/Herdecke University, Witten, Germany; ^2^ Palliative Care Team for Children, University Children’s Hospital, Giessen, Hesse, Germany; ^3^ Department of Anesthesiology, Operative Intensive Care Medicine and Pain Therapy, University Hospital, Giessen, Hesse, Germany; ^4^ Department for Medical Oncology and Palliative Care, University Hospital of Giessen and Marburg, Giessen Site, Germany; ^5^ Institute for Sociology, Technical University of Chemnitz, Chemnitz, Saxony, Germany; ^6^ PedScience Research Institute, Datteln, Germany

**Keywords:** emergency medical service, palliative home care, pediatric emergencies, education, donot-resuscitate order

## Abstract

**Background:**

The prevalence of children with life-limiting conditions (LLCs) is rising. It is characteristic for these children to require 24/7 care. In emergencies, families must decide to call the emergency medical service (EMS) or a palliative care team (PCT)—if available. For EMS teams, an emergency in a child with an LLC is a rare event. Therefore, EMS providers asked for a training unit (TU) to improve their knowledge and skills in pediatric palliative care.

**Aim of the study:**

The questions were as follows: whether a TU is feasible, whether its integration into the EMS training program was accepted, and whether an improvement of knowledge can be achieved.

**Methods:**

We designed and implemented a brief TU based on findings of a previous study that included 1,005 EMS providers. The topics covered were: (1) basics in palliative home care, (2) theoretical aspects, and (3) practical aspects. After participating in the TU, the participants were given a questionnaire to re-evaluate their learning gains and self-confidence in dealing with emergencies in pediatric patients with LLC.

**Results:**

782 (77.8%) of 1,005 participants of the previous study responded to the questionnaire. The average age was 34.9 years (±10.7 years SD), and 75.3% were male. The average work experience was 11.4 years (±9.5 years SD), and 15.2% were medical doctors. We found an increase in theoretical knowledge and enhanced self-confidence in dealing with emergencies in patients with LLC (confidence: before training: 3.3 ± 2.0 SD; after training: 5.7 ± 2.1 SD; min.: 1; max.: 10; *p* < 0.001). The participants changed their approaches to a fictitious case report from more invasive to less invasive treatment. Most participants wanted to communicate directly with PCTs and demanded a standard operating procedure (SOP) for treating patients with LLC. We discussed a proposal for an SOP with the participants.

**Conclusion:**

EMS providers want to be prepared for emergencies in children with LLCs. A brief TU can improve their knowledge and confidence to handle these situations adequately. This TU is the first step to improve collaboration between PCTs and EMS teams.

## Introduction

The prevalence of children with life-limiting conditions (LLCs) has grown significantly in recent decades. In England and Scotland, there was an increase from 26.7 per 10,000 (95% CI, 26.5–27.0) in 2001/2003 to 66.4 per 10,000 (95% CI, 66.0–66.8) in 2017/18 (1). Since the amendment of the German Social Code, members of the statutory health insurance have the right to receive palliative home care (2). This has led to an increasing number of palliative care teams (PCTs), including some for children (3, 4), and more children and adolescents with LLCs are being treated at home (3). Conditions requiring palliative care are diverse and affect children with cancer, heart disease, rare syndromes, trauma sequelae, asphyxia, metabolic diseases, and conditions of other pediatric subspecialties (2, 5, 6). These conditions may be stable but also prone to complications including seizures and pneumonia (7). The number of emergency medical service (EMS) responses for patients with LLCs has also grown (8). Generally, emergencies in infants or children are a challenge for the EMS since they are relatively rare, comprising up to just 10% of all 911 calls (5, 9, 10).

In Australia, EMS providers requested support from pediatric palliative care (PPC) specialists (11). In these situations, besides the complex medical conditions, ethical issues need to be addressed, and immediate decisions must be made (12). The decision by an EMS team and the family to initiate invasive ventilation can have long-term consequences, such as dependence on a respirator or a reduced quality of life. Resuscitation against the will of a patient or their legal representative(s) causes avoidable harm to the patient. If tracheal intubation is performed and mechanical ventilation is started, it is emotionally challenging for a family to end artificial ventilation, even if this is clearly not in the best interest of the child.

Recent publications have focused on the interface between palliative care and the EMS (6, 13–15), but we found very limited data on the specific needs of children and their families.

In a previous study (16), we evaluated the individual experiences of EMS providers with a focus on pediatric emergency calls (8). EMS providers reported distress in these emergencies, suggested invasive treatment (intubation and ventilation) in a case vignette of a child with pneumonia, lacked confidence, and stated the need for specialized pediatric training. In this follow-up study, we report the design, implementation, and results of a 60-minutes training unit (TU) designed to address these issues.

## Methods

### Conception of the TU

**Figure 1:**
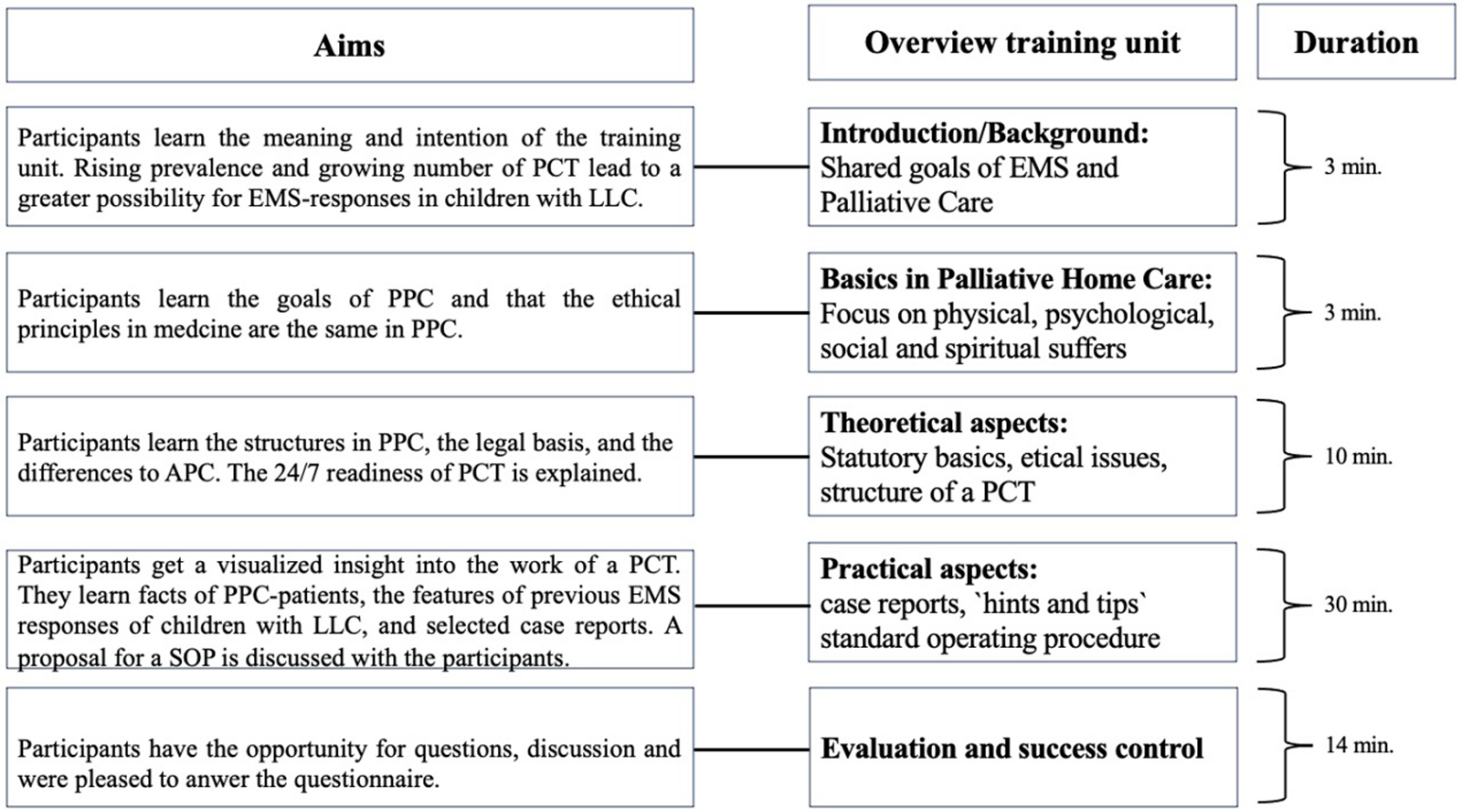
Aims, overview, and duration of the training unit. An actualized version was translated in English and uploaded for cost-free usage (17). APC, adult palliative care; EMS, Emergency Medical Service; LLC, life-limiting condition; PCT, Palliative Care Team; PPC, pediatric palliative care; SOP, standard operating procedure.

We structured the TU based on the demands of an interview study (*n* = 15) and a questionnaire study with 1,005 EMS providers (emergency medical technicians (EMTs)/paramedics, *n* = 789; emergency medical doctors (EMDs), *n* = 226). We identified topics that could be categorized into three main components: basics of palliative care, theoretical and practical aspects of PPC and emergencies (8). The structure, the aims, and the duration of each module in the TU are shown in Figure 1. Following this study, the TU has been translated into English and can be downloaded free of charge (18).

### Integration of the TU into the mandatory training program

In the following step, we conducted meetings with the supervisors of all 10 EMS organizations in Middle Hesse, Germany. In this region with approximately. 1.1 million inhabitants, one PCT is on duty to care for 10–18 PPC patients per day. On average, 6–10 EMS responses are recorded for children with LLCs registered with the PCT per year. Since 2010, EMS providers are required to complete a yearly training program (19) and following the supervisors' decision, the TU was integrated into this compulsory yearly EMS provider training throughout the region.

### Questionnaires (Q1 and Q2)

In the questionnaire of the previous study (Q1), all EMS providers were asked to name topics for a TU and answer questions on their theoretical and practical knowledge of PPC. Additionally, they could describe their experiences of previous EMS responses and their psychological burden. In this study, questionnaire 2 (Q2) was used to compare the results of the participants before the TU—Q1—and afterward—Q2. Both questionnaires are freely accessible in the addendum of this article. All participants were asked to give themselves an individual anonymous code using the second letter of their first name, the second digit of their postal code, the second letter of their place of birth, and the second digit of their mobile number.

### Inclusion of the study participants

All training participants provided informed consent to take part in the Q2 questionnaire. We included participants into the study if they answered both questionnaire Q1 and Q2. All participants had to agree to the study management and data protection (see addendum). All TUs were conducted by the corresponding author in the local EMS training facilities. After the TU was finished, the participants were asked to complete the Q2 questionnaire.

### Evaluation and success control

First, the theoretical and practical knowledge of the EMS providers were examined. Subsequently, the participants were asked to assess a case vignette. In PPC patients, specialized care is required to pursue appropriate goals and actions in accordance with patient preferences. This means invasive treatment is not always wrong. Nevertheless, measures that are not indicated are not an option at any time. The fictitious case was a 15-year-old female with hypoxic-ischemic encephalopathy due to perinatal asphyxia and acute pneumonia and respiratory distress requiring timely intervention to improve gas exchange and/or symptom control. We asked for the participants' therapeutic approach and wanted to know if they would use invasive (e.g., intubation) or non-invasive (e.g., oxygen-inhalation) treatments.

One question was whether the TU could influence the attitude of EMS providers in that typical scenario. In addition, we wanted to know whether the TU met their expectations, whether questions could be asked and were answered sufficiently, and lastly, whether they were interested in repeating this TU and/or would recommend it to colleagues. The questionnaire used a Likert scale from 1 (min. confidence) to 10 (max. confidence).

### Study organization and statistics

Participants could respond in paper form or participate online via a QR code (Lime Survey® GmbH, Hamburg, Germany). The data provided by the study participants were anonymously collected and saved. The individual code generated for each participant excluded the possibility of double participation.

This study was approved by the Ethics Committee of the Justus Liebig University of Giessen, Hesse, Germany (file number: 88/2016). The German Registry of Clinical Studies consented to this study (DRKS-ID: 00013318). Additionally, it was forwarded to the World Health Organization Registry for Clinical Trials.

Descriptive statistics were analyzed using SPSS 26.0 (IBM Corp., NY, USA). To assess group-specific differences, statistical tests were performed. The Wilcoxon test was used to compare ranks if the variables did not follow normal distribution. The Chi-squared test was performed to assess differences in distribution. A *p*-value <0.05 (two-tailed) was considered statistically significant.

## Results

### Demographics

In total, 782 (77.8%) of the previous 1,005 participants of Q1 responded to Q2.

99.2% of the questionnaires were fully completed. Of these, 663 (84.8%) were EMTs and 119 (15.2%) were EMDs (Table 1). Male participants were more common in both groups (EMT: 76%; EMD: 71.4%). EMDs were significantly older and had more work experience than EMTs. The scope of work was different between the two groups; while 74.9% of the EMTs' work was full-time, 50.0% of the EMDs' work was fee-based. All educational levels were represented within the study population (EMT: “Rettungssanitäter” (520 h of education): 25.7%; “Rettungsassistent” (2 years of education): 55.8%, “Notfallsanitäter” (3 years of education): 18.5%) (EMD: residents: 25.2%; fellows: 45.3%; consultants: 22.6%; chiefs: 6.9%).

**Table 1:** Study population/demographics.

	Paramedics (EMT)	Emergency physicians (EMP)	Diff.
Number [*n*/%]	663 (78.5%)	119 (21.5%)	
Gender [*n*/%]	504 (76.0%) ♂	85 (71.4%) ♂	
	MV (0)	MV (1)	
Age (mean ± SD) [years]	33.48 ± 10.4	42.65 ± 9.50	*p* < 0.001
	MV (4)	MV (1)	
Work experience (mean ± SD) [years]	11.02 ± 9.43	14.28 ± 9.63	*p* < 0.001
	MV (6)	MV(1)	
Scope of work [*n*/%]	496 (74.9%)	$\begin{array}{c} 38\;(32.2\text{\%})\\ 19\;(16.1\text{\%})\\ 59\;(50.0\text{\%})\\ 2\;(1.7\text{\%}) \end{array}\}$	*χ*^2^ (3) = 337.9776 *p* < 0.001
Full-time:	148 (22.4%)		
Part-time:	3 (0.5%)		
Fee-based:	15 (2.3%)		
Others:	MV (1)	MV (1)	
Level of education	520-h: 170 (25.7%)	Resident: 30 (25.2%)	
	2-years: 369 (55.8%)	Fellow: 54 (45.4%)	
	3-years: 122 (18.5%)	Consultant: 27 (22.7%)	
	MV (2)	Chief: 6 (5.0%)	
		MV (2)	

MV, missing values; SD, standard deviation; diff., statistical-significant difference; EMT, Emergency Medical Technician/Paramedic; EMD, Emergency Medical Doctor.

1.5% of the study participants of Q2 answered online and directly imported into the database. Most responses were imported manually, and the data were double-checked by the co-authors. Between January 1 and December 31 2018, 78 training units were conducted in 10 facilities with a mean distance of 32.2 km to the PCT (total distance covered: 5,023 km).

### Theoretical and practical knowledge

**Figure 2:**
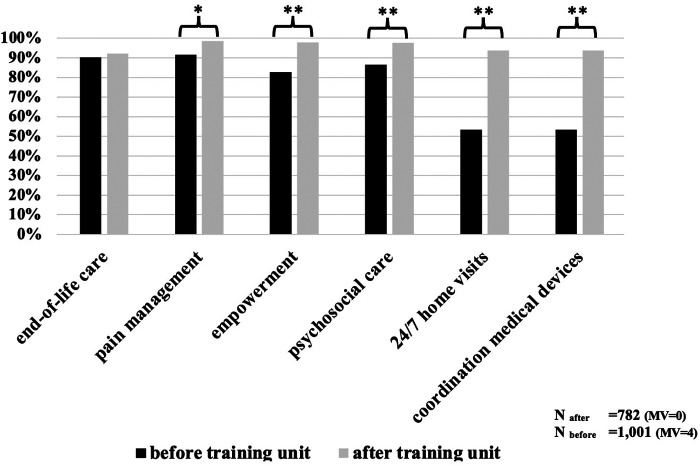
Evaluation of theoretical knowledge before/after training. Bars shows the relation of correct answers. MV, missing values; *p < 0.05; **p < 0.001.

Compared to Q1, we saw an improvement in memorization of theoretical facts (Figure 2 and Table 2). The participants showed a better understanding of the responsibilities of PCTs. In the 12 questions concerning theoretical knowledge, the percentage of correct answers increased from 53%–91% (min.—max.) to 92%–98%. End-of-life care was recognized in the same manner. Participants could better name the legal basis of palliative home care with a significantly increased rate of correct answers from min. 74.4% to max. 98.5%. In Q1, 24.9% of respondents could recognize the typical German abbreviation for a PCT (“SAPV”), and in Q2, the proportion rose to 96.0% (*χ*^2^ = 479.275, *p* < 0.001).

**Table 2:** Evaluation of theoretical/practical knowledge before/after training.

	Correct answers		
	Before training	After training	
Question 27/15	*n* = 1,005 (MV = 0)	*n* = 782 (MV = 0)	***p* < 0.001** **χ^2^ (2) = 128.1143**
“Do you think that adults with statutory health insurance have a legal right to home based palliative care?” (T)	74.5%	96.8%	
Question 28/13	*n* = 982 (MV = 23)	*n* = 782 (MV = 0)	***p* < 0.001** ***χ*^2^ (2) = 181.6097**
“Do you believe that children and adolescents with statutory health insurance have a legal right to home based palliative care?” (T)	73.4%	98.5%	
Question 29/16	*n* = 1,004 (MV = 1)	*n* = 782 (MV = 0)	***p* < 0.001** ***χ*^2^ (2) = 257.2989**
“Do you know the adult palliative care team responsible for your city?” (P)	35.7%	72.8%	
Question 30/14	*n* = 1,004 (MV = 1)	*n* = 782 (MV = 0)	***p* < 0.001** ***χ*^2^ (2) = 1,202.779**
“Do you know the palliative care team for children and adolescents responsible for your city?” (P)	9.3%	91.5%	
Question 31/25	*n* = 997 (MV = 8)	*n* = 782 (MV = 0)	***p* < 0.001** ***χ*^2^ (2) = 271.8298**
“Could you find the contact details of the responsible team (under operational conditions?” (P)	48.9%	85%	

MV, missing values; P, practical question; T, theoretical question.

The bold values are indicate the significance of *p*-value.

We wanted to ascertain whether the participants could find the contact details of the local responsible PCT. In this practical skill, the rate of correct answers increased from 35.7% to 91.5%. Another benefit of the TU was that while only 9.3% knew about the existence of a PPC team beforehand, 91.5% were aware after completing the TU (*χ*^2^ = 382.487, *p* < 0.001).

### Fictitious case report

**Figure 3:**
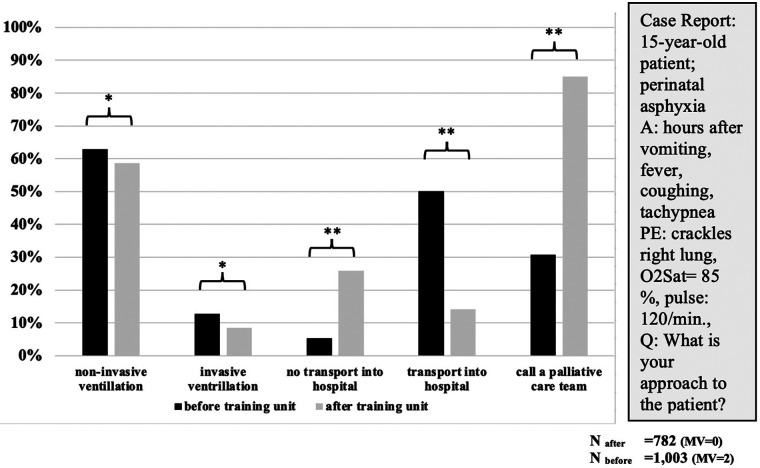
First approaches to case report before/after training (multiple selections possible). A, anamnesis; PE, physical examination; O2Sat, saturation of oxygen; min., minute; Q, question; MV, missing values; *p < 0.05; **p < 0.001.

We saw changes in the approaches to the fictitious but typical case report (Figure 3). There was a slight but significant decline in the rate of selecting invasive ventilation and a reduced rate of transporting the patient to hospital (50% to 14%, *χ*^2^ = 235.879, *p* < 0.001). Before the TU, 30.4% of the participants would have called a PCT in this situation, but afterward, the rate increased to 84.5% (*χ*^2^ = 189.451, *p* < 0.001). No significant differences were seen between EMTs/EMDs or gender.

### Attitude and subjective evaluation

**Figure 4:**
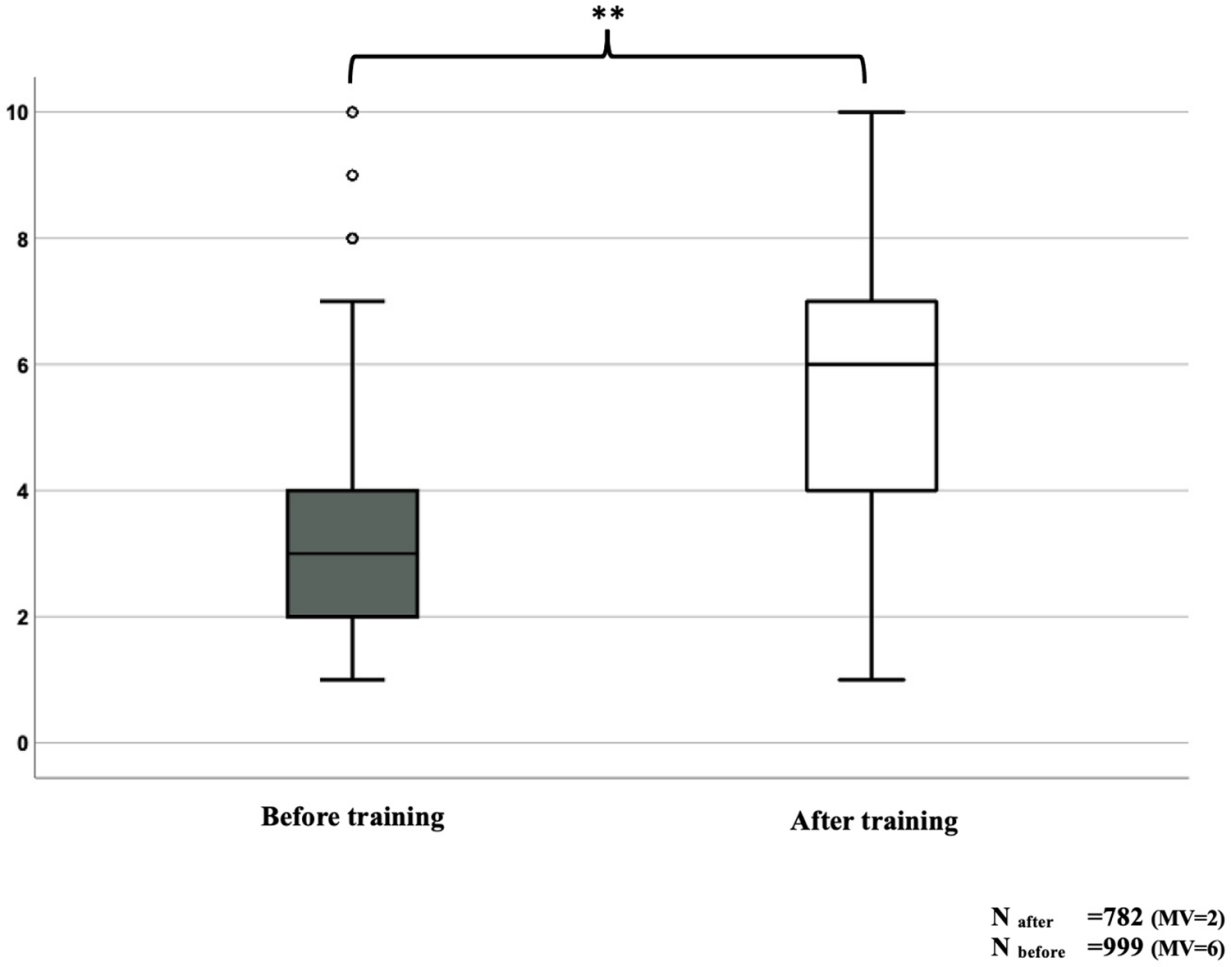
Self-Assessment: Do you feel confident in dealing with emergencies of children under palliative home care? MV, missing values; **p < 0.001.

The participants provided information regarding their confidence in an EMS response to pediatric and adult patients with LLCs (1 = very low to 10 = very high confidence). The confidence with pediatric patients was lower than with adult patients [median 3 vs. median 7; *p* < 0.001 (Wilcoxon’s test)]. We asked whether the respondents felt confident attending future pediatric emergencies following the TU. The participants' confidence increased significantly from a median of 3 to a median of 6 (Figure 4). 97.6% of all participants would recommend the TU to other colleagues. 75.6% stated that they felt better prepared for future responses (EMT: 78.4%; EMD: 60.9%; *χ*^2^ = 17.462, *p* < 0.001).

We asked if participants would put more emphasis on clarifying treatment goals with legal guardians if time permitted. 66.5% (EMT: 61.2% (95% CI, 59.2%–63.0%); EMD: 70.1% (95% CI, 67.4%–73.2%)) were uncertain.

The EMS providers selected how they wanted to receive important information about the patient and communicate with a PCT (Table 3). The patient's emergency file and health-care directive/DNR order were preferred. In addition, there was a significant increase in the belief that this information is already available from the 911 dispatcher.

**Table 3:** Question: how would you prefer to get relevant information about the patient in an emergency? (Before training *N* = 1,002 (MV = 3); after training *N* = 781 (MV = 1)/ (multiple answers possible)).

	911 dispatcher	Patient emergency file	Patients emergency bracelet	Health care directive DNR form	Patients conversation
Before training	56.0%	83.7%	26.4%	21.4%	52.0%
After training	73.1%	93.2%	29.8%	57.7%	55.3%
*p*-value	**<0.001**	**<0.001**	ns	**<0.001**	ns
X^2^	552.695	369.654		2,474.583	

MV, numbers of missing values; ns, not significant; DNR, do-not-resuscitate.

Participants rated the quality of the TU (min. 1 to max. 9 points) with a median of 9 for both EMTs and EMDs (EMT (95% CI, 8.2–8.5); EMD (95% CI, 7.9–8.7). 96.6% of all participants (EMT: 97.2% (95% CI, 96.1%–98.2%); EMD: 93.1% (95% CI, 91.2%–95.5)) answered that all questions were clarified sufficiently.

## Discussion

This study provides the first evaluation of PPC training for EMS providers. In summary, we demonstrated the feasibility and acceptance of a TU designed for this purpose. Besides an increase in theoretical and practical knowledge, there was a slight change in the attitude of EMS providers towards less invasive treatment and more outpatient care alongside collaboration with PCTs. Both PCTs and EMS providers are involved in emergencies of pediatric patients with LLC, and decisions must be made under significant time pressure.

In recent years, publications have indicated a growing awareness of EMS providers regarding patients with LLCs (20–23). Caring for children in emergencies is emotionally demanding, especially in a palliative care setting. Pediatric EMS responses are significantly more overwhelming than emergencies involving adults (24). In an interview study by Eich et al. (25), emergency physicians reported experiencing the greatest deficits in life-threatening emergencies involving children.

Education in palliative care is growing in importance. In the US, 81% of Medicare beneficiaries with cancer visited emergency rooms within six months of the end of life (26). Wang et al. concluded that an educational program adapted to the emergency department must involve the EMS/first responders (13). A study with 182 EMS providers in Georgia revealed that 84.1% had cared for adult patients receiving palliative treatment and only 29.1% reported receiving education for such situations (27). Other authors discuss the need for palliative care training in a framework with public health services (23, 28). All cited studies analyzed aspects of palliative care in emergencies involving adults exclusively. Because of the rising numbers of pediatric patients with LLCs at home and the differences between pediatric and adult palliative care, specific pediatric knowledge should be incorporated in education programs urgently. The TU presented here includes basics and practical aspects of palliative care as required by the National Association of EMS Physicians (NAEMSP) and the American Academy of Hospice and Palliative Medicine (AAHPM) (28).

Our approach of using a 60-minute TU was a compromise. We wanted to provide the participants a concise overview of PPC and have enough time for open questions and discussions—e.g., in small groups. However, another goal was to reach as many EMS providers as possible. The Rescue Service Act of the state of Hesse prescribes 16–38 h of education for EMTs annually, depending on the EMT level. EMDs must complete a minimum of one certified education program (29). The 60-minute duration was easy to integrate into the annual education program. As such, all 10 EMS organizations and all 5 EMD departments could complete the TU.

A strength of our questionnaire studies is the sample size of >1,000 participants (30–33) in Q1, and the relatively high response rate of >70% for Q2, representing all levels of EMT and EMD education and providing a broad spectrum of experience (34). The higher median age of EMDs may be explained by the longer duration of medical studies (6 years) compared to EMT training (520 h to 3 years).

### Theoretical and practical knowledge

In the Q1 study, >90% of providers could recall an EMS response involving adults and 60% had experience with children with an LLC (8). This individual experience could explain the high rate of correct answers regarding the legal basis of palliative home care. It was surprising that only <10% of the study participants were unaware of the existence of a PPC team. In addition to the learning success, the TU could be helpful to improve the network and public perception of PPC.

### Fictitious case report

This case was chosen because it represents a typical EMS scenario in a child receiving palliative home care. This fictitious patient has a life-limiting neurological condition without progression but with progressive sequelae (muscular weakness, dysphagia, aspiration, pneumonia) (16). It was also chosen because there was no right or wrong response as different families may have different preferences. Therefore, for EMS teams, it is best practice to act in accordance with the patients’ and their families’ wishes if there is no obvious violation of the child rights. However, this case has the potential to provide insights into the attitude of the EMS providers. Compared to Q1, there was a significant decrease in invasive treatment and transportation to hospital, which reflects the increased awareness of the EMS providers and the willingness to provide the most comfortable care for the patient. Furthermore, it indicates the need for the EMS to have standards regarding the decision-making processes in emergencies involving patients with LLCs.

### Attitude and subjective evaluation

Regarding the emotional burden of pediatric emergency calls, the providers' confidence was significantly lower than in adult cases. To our knowledge, there are no published studies comparing EMS operations involving children and adults with LLCs. Emotional aspects are important because overload is a factor for developing post-traumatic stress disorder (PTSD) (35). Mishra et al. revealed that in EMS personnel (*n* = 105), 83% reported experiencing symptoms and 5% met the clinical criteria for PTSD (36). Education and peer group meetings may help prevent the development of PTSD (37). A mixed methods study by Shearer that included 66 participants in Perth, Australia, found that EMS personnel sought further education in communication and ethics to improve their confidence (38). Although emotional health was not assessed in our research questions, we suggest that the mental health of EMS providers regarding emergencies in children with LLCs should be further investigated.

Another result of our study was that before and after the TU, most participants felt responsible for patients with LLCs in emergencies even if they were treated by a 24/7 palliative home care team. This is consistent with the work of Hoare in Cambridge, UK, where study participants were broadly consistent in allowing adults to die at home (39). In a study from Canada, Jensen discussed a program for paramedics who care for long-term care residents to be able to play a key role in end-of-life care (40).

**Figure 5:**
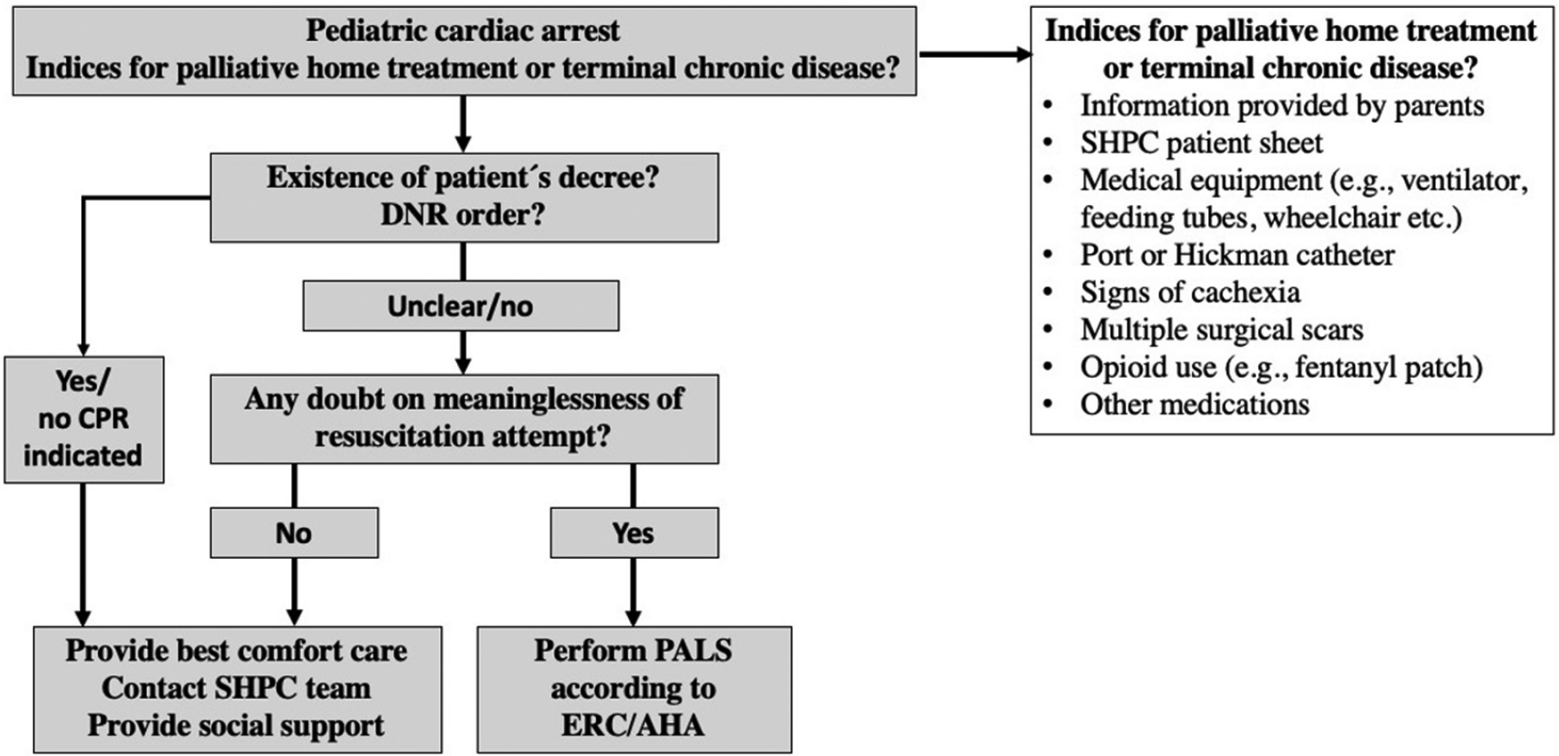
Proposal of an Algorithm for the management of cardiac arrest in pediatric patients under home palliative therapy (41). AHA, American Heart Association; DNR, Do not resuscitate; ERC, European Resuscitation Council; PALS, Pediatric advanced life support; SHPC, Specialized palliative home care.

One major issue discussed with the participants of Q2 was both EMTs’ and EMDs' fear of making a mistake. A family's journey from hoping their beloved child will survive to accepting the unacceptable is very complex and may be difficult for an EMS team to understand, especially within a few minutes. However, in emergencies, there is a need for rapid decision-making. Therefore, we proposed a standard operating procedure (SOP) in the TU (Figure 5). We had three main aims with this proposal: (a) to encourage the EMS providers to act directly if there is no information that allows withholding of CPR; (b) to encourage collaboration with a PCT; and (c) to recognize a child with an LLC. These may be difficult to do alongside taking an anamnesis with the parents, which, in practice, is not only limited by the time pressure but also by possible language or cultural barriers. In the literature, we found no instructions or SOP for pediatric patients with LLCs, and during the discussions in the TU, the proposed SOP appeared to be broadly accepted.

All patients of a PCT and their families should be provided with a comprehensible EMS letter, an emergency folder (24/7 contact number of the PCT, medication plan), and if available, a health-care directive (42). If an emergency is anticipated, the legal guardians should be fully informed about therapeutic options and their consequences. If they have a clear treatment preference, they should be offered an advance directive to sign on behalf of their child. Communication, information, and clarity of the therapeutic goal were the issues that were most frequently and comprehensibly asked about after the TU. The data from previous EMS responses to children with LLCs were helpful to underpin the fact that in most cases, there is enough time to ask for the emergency folder, call the PCT, and make a decision for the child together with the family. If CPR is not contraindicated and the parents don’t disagree, then advanced life-support (ALS) should be started until the EMS team gets new information. DNR orders or Physician Orders for Life-Sustaining Treatment (POLST) forms can help the EMS make decisions (43).

In time-critical situations, it is important that the EMS is rapidly available despite the 24/7 on-call PCT, which needs more time to arrive at the scene. Cooperation at the important interface of EMS providers and PCTs is urgently needed for this highly vulnerable group of children and adolescents. The TU was a further step to improve this cooperation.

### Limitations of the study

The questionnaire was developed with a focus on practical aspects, but there was no Delphi process to review the questions and no tests were performed to assess reliability or validity.

Critically, it should be mentioned that the TU was only led by one trainer, which may have had a negative impact on objectivity and generalizability.

The recruitment rate could have been improved. The differing requirements of 16–38 h of annual training for EMTs might have contributed to the fact that we did not reach all 431 EMTs with 520 h or 2 years of education from Q1.

The 60-minute duration of the TU was too short to cover all the important issues of palliative care. On the other hand, this duration made it easy to implement the TU into the mandatory education program of all 10 EMS organizations.

This study is limited to the experiences of German EMS teams with a system involving prehospital EMDs.

We decided to ask the participants to answer the questionnaire immediately after the TU to avoid a low response rate. This, however, does not allow conclusions regarding long-term effects of the TU.

Fictitious case reports cannot represent reality, but they are an appropriate tool to conduct studies in palliative care (23, 34). We chose a vignette that was, according to our previous study, typical for an EMS response involving children with LLCs (16).

To further increase the scientific validity, a control group and different trainers should be used in subsequent studies.

## Conclusions

The EMS plays a role in out-of-hospital PPC. Training EMS providers is a valuable approach to improving collaboration between EMS teams and PCTs. A practical, comprehensible TU on PPC for EMS providers can increase knowledge and confidence in these rare and challenging responses. A TU might improve understanding at this important interface for children with LLCs.

## Data Availability

The original contributions presented in the study are included in the article/**Supplementary Material**, further inquiries can be directed to the corresponding author.
