# Supplementary material for: Implementation and evaluation of a palliative care training unit for EMS providers
Source: Front Pediatr. 2023 Sep 27;11:1272706. doi: 10.3389/fped.2023.1272706 (PMC10565227; doi:10.3389/fped.2023.1272706)
Supplement: Supplementary file 1 [file Datasheet1.pdf]

Questionnaire 1

1. How old are you?
Please enter your answer here:

[ ]

Figures in years

2. Gender
Please select only one of the following answers:

○ female ○ male

3. Where do you work?
Please select only one of the following answers:

○ Giessen

○ Marburg

○ Lahn-Dill-Kreis

○ Limburg-Weilburg

○ Vogelsberg

4. Which ambulance service organisation do you work for?
Please select only one of the following answers:

○ German Red Cross

○ Malteser Hilfsdienst

○ Fire brigade

○ Arbeiter-Samariter-Bund (Workers' Samaritan Federation)

○ Johanniter

○ private

○ Other

5. What professional group do you belong to?
Please select only one of the following answers:

○ Doctor ○ Non-doctor

6. What is your employment relationship like?

Please select only one of the following answers:

○ full-time

○ part-time/part-time

○ Fee basis

○ Other

7. How many years of work experience do you have?

Please enter your answer here:

[ ]

Only numbers may be entered in this field.

8. Degree of education

Please select only one of the following answers:

○ Intern

○ Specialist

○ Attending

○ Head physician

○ Paramedic (520 h)

○ Paramedic 2-years

○ Emergency Paramedic 3-years

9. For non-doctors: Do you have extended care measures (EVM) Hesse?

Please select all applicable answers:

☐ Intubation

☐ i.v. access

☐ administer drugs

☐ Other: ____________

10. Have you ever had to care for a child/adolescent with a life-limiting illness as part of your rescue work?

Please select only one of the following answers:

○ Yes ○ No ○ I don't know

11. If so, what care did you provide/what can you remember?
Please select all applicable answers:

☐ Driving to the hospital

☐ Reanimation

☐ Staying at home

☐ Handover to the family doctor/emergency medical service

☐ patient was deceased.

☐ tactical problems / delays

☐ Other:___________________________________

12. If so, would you briefly describe what the situation was like for you?
Please enter your answer here:

13. Did you find the assignment stressful?
Please select only one of the following answers:

○ Yes ○ No

14. If so, how much did the assignment burden you?
Please select the appropriate answer for each point: (1 = low stress 10 = very stressful)

	1	2	3	4	5	6	7	8	9	10
Severity of the burden	○	○	○	○	○	○	○	○	○	○

15. Have you ever had contact with a palliative care team for children and adolescents in an emergency?
Please select only one of the following answers:

○ And ○ No

16. Do you feel up to a palliative situation with children and adolescents in the field?
Please select only one of the following answers:

○ Yes ○ No ○ I don't know

17. Have you ever had to care for an adult with a life-limiting illness as part of your emergency medical service work?
Please select only one of the following answers:

○ Yes ○ No ○ I don't know

18. If so, what care did you provide?
Please select all applicable answers:

☐ Driving to the hospital

☐ Reanimation

☐ Staying at home

☐ Handover to family doctor/medical emergency service

☐ Deceased

☐ tactical problems / delays

19. If so, would you briefly describe what the situation was like for you?

Please enter your answer here:

20. Did you find this assignment stressful?
Please select only one of the following answers:

○ Yes ○ No

21. If so, how much did the assignment burden you?
Please select the appropriate answer for each point: (1 = low stress 10 = very stressful)

	1	2	3	4	5	6	7	8	9	10
Severity of the burden	○	○	○	○	○	○	○	○	○	○

22. Have you ever had contact with an adult palliative care team in an emergency situation?
Please select only one of the following answers:

○Yes ○No

23. Do you feel up to a palliative situation with adults in the field?

Please select only one of the following answers:

○Yes ○No ○I don't know

24. Imagine the following situation: You are called to an emergency response (with RTW/NEF/NAW). Operational keyword: "Emergency with child"! They arrive at the scene and find a 15-year-old. The patient has multiple disabilities due to an infant lack of oxygen. The patient suffers from cough and fever after an episode of vomiting. The patient suffers from dyspnea and the oxygen saturation on the pulse oximeter shows 85%. The family doctor cannot be reached. How do you proceed after the usual measures (medical history/physical examination/vital sign check)?

Please select all applicable answers:

☐invasive ventilation

☐non-invasive ventilation

☐fast transport to the clinic

☐Leave the patient at home

☐Palliative sedation

☐if possible, call a palliative care team

☐ Other:____________________

25. Do you know the abbreviation SAPV?
Please select only one of the following answers:

○Yes ○No

26. If so, what does the above abbreviation stand for?
Please enter your answer here:

27. If so, do you believe that adults with statutory health insurance have a legal right to a SAPV?
Please select only one of the following answers:

○Yes ○No ○I don't know

28. If so, do you believe that children and adolescents with statutory health insurance have a legal right to a SAPV?
Please select only one of the following answers:

○Yes ○No ○I don't know

29. Do you know the adult palliative care team responsible for you?
Please select only one of the following answers:

○Yes ○No

30. Do you know the palliative care team for children and adolescents responsible for you?
Please select only one of the following answers:

○Yes ○No

31. Could you find the contact details of the responsible team (adults and/or children) under operational conditions?
Please select only one of the following answers:

○Yes ○No

32. What are the tasks of palliative care teams?
Please select all applicable answers:

☐End-of-life care

☐Pain

☐Symptom control

☐Avoidance of hospitalization

☐Stabilization of the family environment

☐psychosocial care

☐Supply of assistive devices

☐Emergency management

33. Do you also feel responsible for the care of adult or child palliative patients in emergency situations?
Please select only one of the following answers:

○ Yes ○ No ○ I don't know

34. How would you prefer to be able to contact the responsible palliative care team in real life?
Please select all applicable answers:

☐ The control center is informed in advance about the palliative situation (e.g. by fax)

☐ Emergency folder with hotline number at the patient's premises

☐ Bracelet at the patient

☐ Emergency doctor's letter available

☐ I ask the patient/relatives themselves.

35. As part of the normal annual training events in emergency medical services/emergency medicine, would you like to see a section on palliative care as an interface to prehospital emergency medicine?
Please select only one of the following answers:

○ Yes ○ No ○ I don't know

36. If so, what is the maximum length of such training?
Please enter your answer here:

[ ]

Expressed in minutes

37. Would you like it if the pediatric palliative care team responsible for you presented itself to you at the guard/hospital/control center?
Please select only one of the following answers:

○ Yes ○ No ○ I don't know

Since we want to offer you further training from the results of this survey, we would like to ask you again about a possible participation. It would be beneficial to compare the current results with the original data. For this purpose, an assignment in non-named form would be helpful (so-called pseudonymization). Finally, we ask you to give yourself a code that cannot be linked to your identity.

Please fill in the following column:

1. __ (second letter of the first name) 2. __ (second digit of one's own postal code)

3. __ (second letter of place of birth) 4. __ (second digit of the mobile number)

Thank you for your support!
