# Supplementary material for: Implementation and evaluation of a palliative care training unit for EMS providers
Source: Front Pediatr. 2023 Sep 27;11:1272706. doi: 10.3389/fped.2023.1272706 (PMC10565227; doi:10.3389/fped.2023.1272706)
Supplement: Supplementary file 2 [file Datasheet2.pdf]

Questionnaire 2

1. How old are you?

Please enter your answer here:

Figures in years

2. Gender

Please select only one of the following answers:

○ female ○ male

3. Where do you work?

Please select only one of the following answers:

○ Giessen

○ Marburg

○ Lahn-Dill-Kreis

○ Limburg-Weilburg

○ Vogelsberg

4. Which ambulance service organisation do you work for?

Please select only one of the following answers:

○ German Red Cross

○ Malteser

○ Fire brigade

○ Arbeiter-Samariter-Bund (Workers' Samaritan Federation)

○ Johanniter

○ private

○ Other

5. What professional group do you belong to?

Please select only one of the following answers:

○ Doctor ○ Non-doctor

6. What is your employment relationship like?
Please select only one of the following answers:

○ full-time

○ part-time/part-time

○ Fee basis

○ Other

7. How will you be deployed?
Please select all applicable answers:

☐ KTW (ambulance)

☐ RTW (ambulance)

☐ MZF (Multi-Purpose Vehicle)

☐ RTH/ITH (Rescue Helicopter/Intensive Care Transport Helicopter)

☐ Control Center

☐ NAW/NEF (ambulance/ambulance)

8. How many years of work experience do you have?

Please enter your answer here:

Only numbers may be entered in this field.

9. Degree of education
Please select only one of the following answers:

○ Intern

○ Specialist

○ Attending

○ Head physician

○ Paramedic (520 h)

○ Paramedic (2-years)

○ Emergency Paramedic (3-years)

10. For non-doctors: Do you have extended care measures (EVM) Hesse?

Please select all applicable answers:

☐Intubation

☐i.v. access

☐administer drugs

☐ Other: ____________

11. Do you know the abbreviation SAPV?

Please select only one of the following answers:

○Yes ○No

12. If so, what does the above abbreviation stand for?

Please enter your answer here:

13. Do you believe that children and adolescents with statutory health insurance have a legal right to a SAPV?

Please select only one of the following answers:

○Yes ○No ○I don't know

14. Do you know the palliative care team for children and adolescents responsible for you?

Please select only one of the following answers:

○Yes ○No

15. Do you believe that adults with statutory health insurance have a legal right to a SAPV?

Please select only one of the following answers:

○Yes ○No ○I don't know

16. Do you know the adult palliative care team responsible for you?

Please select only one of the following answers:

○Yes ○No

17. What are the tasks of palliative care teams?

Please select all applicable answers:

☐End-of-life care

☐Pain

☐Symptom control

☐Avoidance of hospitalization

☐Stabilization of the family environment

☐psychosocial care

☐Supply of assistive devices

☐Emergency management

18. Do you also feel responsible for the care of adult or child palliative patients in emergency situations?

Please select only one of the following answers:

○Yes ○No ○I don't know

19. How would you prefer to be able to contact the responsible palliative care team in real life?

Please select all applicable answers:

☐The control centre is informed in advance about the palliative situation (e.g. by fax)

☐Emergency folder with hotline number at the patient's premises

☐Bracelet at the patient

☐Emergency doctor's letter available

☐I ask the patient/relatives themselves.

20. How much do you feel up to a palliative situation with a d u l t s in the field?

Please select the correct answer for each point: (1 = very uncertain 10 = very certain)

	1	2	3	4	5	6	7	8	9	10
Safety	○	○	○	○	○	○	○	○	○	○

21. How much do you feel up to a palliative situation with children or c h I l d r e n in the field?

Please select the correct answer for each point: (1 = very uncertain 10 = very certain)

	1	2	3	4	5	6	7	8	9	10
Safety	○	○	○	○	○	○	○	○	○	○

22. How confident did you feel before the training in dealing with pediatric palliative patients?
Please select the correct answer for each point: (1 = very uncertain 10 = very certain)

	1	2	3	4	5	6	7	8	9	10
Safety	◯	◯	◯	◯	◯	◯	◯	◯	◯	◯

23. How confident do you feel in dealing with pediatric palliative patients after the training?
Please select the correct answer for each point: (1 = very uncertain 10 = very certain)

	1	2	3	4	5	6	7	8	9	10
Safety	◯	◯	◯	◯	◯	◯	◯	◯	◯	◯

24. Now, after the training, can you find the contact details of the team responsible for c h I l d r e n under operational conditions?
Please select only one of the following answers:

◯Yes ◯No

25. Now, after the training, can you find the contact details of the team responsible for c h I l d r e n under operational conditions?
Please select only one of the following answers:

◯Yes ◯No

26. Did you find the training helpful to be able to apply it practically in the possible operational situation?
Please select only one of the following answers:

◯Yes ◯No

27. If so, how helpful did you find the training?
Please select the appropriate answer for each point: (1 = not very helpful 10 = very helpful)

	1	2	3	4	5	6	7	8	9	10
Learning Effect	◯	◯	◯	◯	◯	◯	◯	◯	◯	◯

28. Imagine the following situation:

You will be called to an emergency response (with RTW/NEF/NAW). Operational keyword: "Emergency with child"! They arrive at the scene and find a 19-year-old young adult. The young man has multiple disabilities due to an early childhood lack of oxygen (after a drowning accident). The patient suffers from cough and fever. It shows clear orthopnea and the oxygen saturation on the pulse oximeter shows 82%. The family doctor cannot be reached. How do you proceed according to the usual measures (medical history/physical examination/vital sign check)?

Please select all applicable answers:

☐invasive ventilation

☐non-invasive ventilation

☐fast transport to the clinic

☐Leave the patient at home

☐Palliative sedation

☐if possible, call a palliative care team

☐ Other:____________________

29. Would you recommend your colleagues to participate in this training?

Please select only one of the following answers:

○Yes ○No ○I don't know

30. Did you have ample opportunity to ask questions?

Please select only one of the following answers:

○Yes ○No ○I don't know

31. Have the questions been answered comprehensively?

Please select only one of the following answers:

○Yes ○No ○I don't know

32. Has your opinion on outpatient palliative care changed after the presentation?

○Yes ○No ○I don't know

33. What do you think has changed in concrete terms?

Please select all applicable answers:

☐ I now know the tasks of palliative care teams better.

☐ I know how to reach the children's palliative care team.

☐ I know how to reach the adult palliative care team that is responsible for me.

☐ This training helps me to better cope with future operations.

☐ This training was not helpful.

☐ Other:____________________

34. What applies to you personally? After this training, I would be more likely to be able to diagnose life-threatening emergencies with palliative patients

Please select only one of the following answers:

○ prone to resuscitation with invasive measures.

○ tend not to resuscitation, but to passive, symptomatic action.

○ I don't know

35. Is there anything else you would like to tell us?

Since we want to compare the results of this survey with the results before the training, we finally ask you to give yourself a code that cannot be linked to your identity (so-called pseudonymization).

Please fill in the following column:

1. __ (second letter of the first name) 2. __ (second digit of one's own postal code)
3. __ (second letter of place of birth) 4. __ (second digit of the mobile number)

Thank you for your support!
